# Bis(2-hydroxy­benzaldehyde oxime) *O*,*O*′-butane-1,4-diyldicarbonyl ether

**DOI:** 10.1107/S1600536809016663

**Published:** 2009-05-20

**Authors:** Bijan Etemadi, Reza Kia, Hashem Sharghi, Mona Hosseini Sarvari

**Affiliations:** a Faculty of Sciences, Department of Earth Sciences, Shiraz University, Shiraz, 71454, Iran; b Faculty of Sciences, Chemistry Department, Shiraz University, Shiraz, 71454, Iran

## Abstract

The mol­ecule of the title compound, C_20_H_20_N_2_O_6_, lies across a crystallographic inversion centre, the asymmetric unit comprising one half-mol­ecule. An intra­molecular O—H⋯N hydrogen bond generates a six-membered ring, producing an *S(6)* ring motif. Pairs of inter­molecular C—H⋯O hydrogen bonds link neighbouring mol­ecules into a layer with *R* ^2^_2_(38) ring motif. The crystal structure is further stabilized by the inter­molecular C—H⋯π inter­actions.

## Related literature

For bond-length data, see Allen *et al.* (1987 ▶). For hydrogen bond motifs, see Bernstein *et al.* (1995 ▶). For Schiff bases, see: Granovski *et al.*, (1993 ▶). For the synthesis, see: Hosseini Sarvari (2003 ▶).
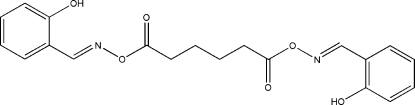


## Experimental

### 

#### Crystal data

C_20_H_20_N_2_O_6_
*M* *_r_* = 384.38
Monoclinic, 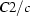

*a* = 13.0293 (11) Å
*b* = 5.5464 (4) Å
*c* = 25.538 (2) Å
β = 91.348 (7)°
*V* = 1845.0 (3) Å^3^
*Z* = 4
Mo *K*α radiation
μ = 0.10 mm^−1^
*T* = 120 K
0.45 × 0.11 × 0.10 mm

#### Data collection

STOE IPDSII diffractometer
Absorption correction: numerical (*X-RED32*; Stoe & Cie (2005 ▶) *T*_min_ = 0.956, *T*_max_ = 0.985
10357 measured reflections
2493 independent reflections
2098 reflections with *I* > 2σ(*I*)
*R*_int_ = 0.046

#### Refinement

*R*[*F* ^2^ > 2σ(*F* ^2^)] = 0.047
*wR*(*F* ^2^) = 0.099
*S* = 1.10
2493 reflections
135 parameters
H atoms treated by a mixture of independent and constrained refinement
Δρ_max_ = 0.31 e Å^−3^
Δρ_min_ = −0.20 e Å^−3^

### 

Data collection: *X-AREA* (Stoe & Cie, 2005 ▶); cell refinement: *X-AREA*; data reduction: *X-AREA*; program(s) used to solve structure: *SHELXTL* (Sheldrick, 2008 ▶); program(s) used to refine structure: *SHELXTL*; molecular graphics: *SHELXTL*; software used to prepare material for publication: *SHELXTL* and *PLATON* (Spek, 2009 ▶).

## Supplementary Material

Crystal structure: contains datablocks global, I. DOI: 10.1107/S1600536809016663/at2778sup1.cif

Structure factors: contains datablocks I. DOI: 10.1107/S1600536809016663/at2778Isup2.hkl

Additional supplementary materials:  crystallographic information; 3D view; checkCIF report

## Figures and Tables

**Table 1:** Hydrogen-bond geometry (Å, °)

*D*—H⋯*A*	*D*—H	H⋯*A*	*D*⋯*A*	*D*—H⋯*A*
O1—H1⋯N1	0.91 (3)	1.79 (3)	2.5836 (15)	144 (2)
C4—H4⋯O1^i^	0.93	2.45	3.1874 (17)	136
C2—H2⋯*Cg*1^ii^	0.93	2.75	3.4659 (14)	134

Symmetry codes: (i) 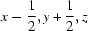; (ii) 
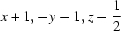
. *Cg*1 is the centroid of the C1–C6 benzene ring.

**Fig. 1.**
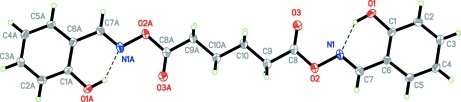
The molecular structure of the title compound, showing 50% probability displacement ellipsoids and the atomic numbering. Intramolecular hydrogen bonds are shown as dashed lines. Symmetry code for A suffix: -x + 1/2, -y + 1/2, -z + 1].

**Fig. 2.**
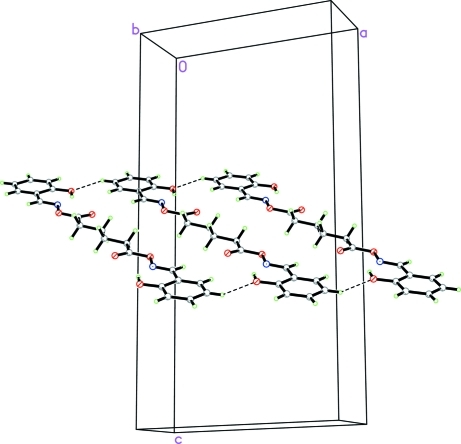
The crystal packing of the title compound, showing linking of the molecules into a layer through R22(38) motifs. Intermolecular interactions are drawn as dashed lines.

## supplementary crystallographic information

### Comment

Schiff base compounds are some of the most important stereochemical models in transition metal coordination chemistry, with their ease of preparation and structural variations (Granovski *et al.*, 1993). In continuation of our works on the synthesis and structural characterization of Schiff base ligands here we report the structure of the title compound.

The asymmetric unit of the title compound, Fig. 1, lies across a crystallographic inversion centre. Intramolecular O—H···N hydrogen bond generates a six-membered ring, producing an *S(6)* ring motif (Bernstein *et al.*, 1995). Pairs of intermolecular C—H···O hydrogen bonds link neighbouring molecules into a layer with *R^2^_2_(38)* ring motif (Fig. 2). The crystal structure is further stabilized by the intermolecular C—H···π interactions [*Cg*1 is the centroid of the C1–C6 benzene ring] (Table 1).

### Experimental

An ethyl acetate solution (40 ml) of salicylaldoxime (2 mmol, 768 mg) was treated with butanedicarboxylic acid chloride (1 mmol, 183 mg) at -5 ^0^ C. The mixture was stirred for 30 min and then filtered and the resulting white powder dried under air (Hosseini Sarvari, 2003). Single crystals suitable for *X*-ray diffraction were obtained from an ethanol solution.

### Refinement

The O-bound and C7 bound hydrogen atoms were located from the difference Fourier map and refined freely. The rest of the hydrogen atoms were positioned geometrically [C—H = 0.93–97 Å] and refined using a riding model approximation with U_iso_(H) = 1.2U_eq_(C).

### Crystal data

**Table:**

C_20_H_20_N_2_O_6_	*F*(000) = 808
*M**_r_* = 384.38	*D*_x_ = 1.384 Mg m^−^^3^
Monoclinic, *C*2/*c*	Mo *K*α radiation, λ = 0.71073 Å
Hall symbol: -C 2yc	Cell parameters from 1302 reflections
*a* = 13.0293 (11) Å	θ = 3.1–29.2°
*b* = 5.5464 (4) Å	µ = 0.10 mm^−^^1^
*c* = 25.538 (2) Å	*T* = 120 K
β = 91.348 (7)°	Needle, colourless
*V* = 1845.0 (3) Å^3^	0.45 × 0.11 × 0.10 mm
*Z* = 4	

### Data collection

**Table:**

STOE IPDS II diffractometer	2493 independent reflections
Radiation source: fine-focus sealed tube	2098 reflections with *I* > 2σ(*I*)
graphite	*R*_int_ = 0.091
Detector resolution: 0.15 pixels mm^-1^	θ_max_ = 29.2°, θ_min_ = 3.1°
rotation method scans	*h* = −17→17
Absorption correction: numerical (*X-RED32*; Stoe & Cie (2005)	*k* = −7→7
*T*_min_ = 0.956, *T*_max_ = 0.985	*l* = −35→34
41515 measured reflections	

### Refinement

**Table:**

Refinement on *F*^2^	Primary atom site location: structure-invariant direct methods
Least-squares matrix: full	Secondary atom site location: difference Fourier map
*R*[*F*^2^ > 2σ(*F*^2^)] = 0.047	Hydrogen site location: inferred from neighbouring sites
*wR*(*F*^2^) = 0.099	H atoms treated by a mixture of independent and constrained refinement
*S* = 1.10	*w* = 1/[σ^2^(*F*_o_^2^) + (0.0425*P*)^2^ + 1.8216*P*] where *P* = (*F*_o_^2^ + 2*F*_c_^2^)/3
2493 reflections	(Δ/σ)_max_ = 0.004
135 parameters	Δρ_max_ = 0.31 e Å^−^^3^
0 restraints	Δρ_min_ = −0.20 e Å^−^^3^

### Special details

**Table:**

Experimental. The low-temperature data was collected with the Oxford Cyrosystem Cobra low-temperature attachment.
Geometry. All esds (except the esd in the dihedral angle between two l.s. planes) are estimated using the full covariance matrix. The cell esds are taken into account individually in the estimation of esds in distances, angles and torsion angles; correlations between esds in cell parameters are only used when they are defined by crystal symmetry. An approximate (isotropic) treatment of cell esds is used for estimating esds involving l.s. planes.
Refinement. Refinement of F^2^ against ALL reflections. The weighted R-factor wR and goodness of fit S are based on F^2^, conventional R-factors R are based on F, with F set to zero for negative F^2^. The threshold expression of F^2^ > 2sigma(F^2^) is used only for calculating R-factors(gt) etc. and is not relevant to the choice of reflections for refinement. R-factors based on F^2^ are statistically about twice as large as those based on F, and R- factors based on ALL data will be even larger.

### Fractional atomic coordinates and isotropic or equivalent isotropic displacement parameters (Å^2^)

**Table:**

	*x*	*y*	*z*	*U*_iso_*/*U*_eq_	
C1	0.79600 (9)	−0.3740 (2)	0.32698 (5)	0.0186 (3)	
C2	0.73433 (10)	−0.5276 (2)	0.29667 (5)	0.0204 (3)	
H2	0.7620	−0.6677	0.2828	0.025*	
C3	0.63188 (10)	−0.4721 (2)	0.28720 (5)	0.0206 (3)	
H3	0.5909	−0.5768	0.2674	0.025*	
C4	0.58953 (9)	−0.2610 (3)	0.30699 (5)	0.0212 (3)	
H4	0.5207	−0.2245	0.3004	0.025*	
C5	0.65095 (9)	−0.1061 (2)	0.33650 (5)	0.0192 (3)	
H5	0.6230	0.0355	0.3495	0.023*	
C6	0.75482 (9)	−0.1590 (2)	0.34720 (5)	0.0168 (2)	
C7	0.81678 (10)	0.0064 (2)	0.37953 (5)	0.0193 (3)	
H7	0.7866 (13)	0.154 (3)	0.3912 (7)	0.027 (4)*	
C8	1.05836 (10)	0.0434 (2)	0.43627 (5)	0.0204 (3)	
C9	1.11260 (10)	0.2321 (2)	0.46898 (5)	0.0208 (3)	
H9A	1.0741	0.2617	0.5004	0.025*	
H9B	1.1155	0.3816	0.4494	0.025*	
C10	1.22133 (10)	0.1530 (2)	0.48435 (5)	0.0206 (3)	
H10B	1.2588	0.1167	0.4529	0.025*	
H10A	1.2181	0.0069	0.5051	0.025*	
N1	0.90918 (8)	−0.0542 (2)	0.39115 (4)	0.0224 (3)	
O1	0.89508 (7)	−0.4424 (2)	0.33553 (4)	0.0289 (3)	
H1	0.9268 (18)	−0.333 (5)	0.3572 (10)	0.062 (7)*	
O2	0.96033 (7)	0.11970 (18)	0.42331 (4)	0.0223 (2)	
O3	1.09270 (8)	−0.14750 (19)	0.42343 (4)	0.0271 (2)	

### Atomic displacement parameters (Å^2^)

**Table:**

	*U*^11^	*U*^22^	*U*^33^	*U*^12^	*U*^13^	*U*^23^
C1	0.0151 (5)	0.0196 (6)	0.0210 (6)	0.0028 (4)	−0.0008 (4)	0.0010 (5)
C2	0.0222 (6)	0.0177 (6)	0.0213 (6)	0.0026 (5)	0.0002 (5)	−0.0012 (5)
C3	0.0191 (6)	0.0219 (6)	0.0207 (6)	−0.0031 (5)	−0.0022 (4)	−0.0009 (5)
C4	0.0146 (5)	0.0261 (7)	0.0227 (6)	0.0016 (5)	−0.0013 (4)	−0.0001 (5)
C5	0.0172 (6)	0.0199 (6)	0.0204 (6)	0.0031 (5)	0.0011 (4)	−0.0008 (5)
C6	0.0163 (5)	0.0171 (6)	0.0170 (5)	−0.0003 (4)	−0.0008 (4)	0.0010 (5)
C7	0.0202 (6)	0.0185 (6)	0.0191 (6)	−0.0007 (5)	−0.0006 (4)	−0.0001 (5)
C8	0.0194 (6)	0.0224 (7)	0.0192 (6)	−0.0037 (5)	−0.0019 (4)	0.0009 (5)
C9	0.0209 (6)	0.0207 (6)	0.0206 (6)	−0.0033 (5)	−0.0025 (5)	−0.0011 (5)
C10	0.0204 (6)	0.0216 (6)	0.0197 (6)	−0.0032 (5)	−0.0035 (5)	−0.0012 (5)
N1	0.0197 (5)	0.0231 (6)	0.0241 (6)	−0.0047 (4)	−0.0043 (4)	−0.0052 (5)
O1	0.0170 (5)	0.0292 (6)	0.0402 (6)	0.0085 (4)	−0.0070 (4)	−0.0111 (5)
O2	0.0199 (5)	0.0217 (5)	0.0250 (5)	−0.0030 (4)	−0.0048 (4)	−0.0048 (4)
O3	0.0250 (5)	0.0231 (5)	0.0330 (5)	0.0006 (4)	−0.0067 (4)	−0.0060 (4)

### Geometric parameters (Å, °)

**Table:**

C1—O1	1.3583 (15)	C7—H7	0.958 (18)
C1—C2	1.3935 (18)	C8—O3	1.1985 (17)
C1—C6	1.4105 (18)	C8—O2	1.3784 (16)
C2—C3	1.3857 (17)	C8—C9	1.5047 (18)
C2—H2	0.9300	C9—C10	1.5255 (18)
C3—C4	1.3943 (19)	C9—H9A	0.9700
C3—H3	0.9300	C9—H9B	0.9700
C4—C5	1.3850 (18)	C10—C10^i^	1.526 (2)
C4—H4	0.9300	C10—H10B	0.9700
C5—C6	1.4054 (17)	C10—H10A	0.9700
C5—H5	0.9300	N1—O2	1.4226 (14)
C6—C7	1.4639 (17)	O1—H1	0.91 (3)
C7—N1	1.2782 (17)		
			
O1—C1—C2	116.85 (12)	C6—C7—H7	119.1 (10)
O1—C1—C6	123.07 (12)	O3—C8—O2	123.73 (12)
C2—C1—C6	120.08 (11)	O3—C8—C9	126.36 (12)
C3—C2—C1	120.10 (12)	O2—C8—C9	109.91 (11)
C3—C2—H2	120.0	C8—C9—C10	111.35 (11)
C1—C2—H2	120.0	C8—C9—H9A	109.4
C2—C3—C4	120.75 (12)	C10—C9—H9A	109.4
C2—C3—H3	119.6	C8—C9—H9B	109.4
C4—C3—H3	119.6	C10—C9—H9B	109.4
C5—C4—C3	119.31 (11)	H9A—C9—H9B	108.0
C5—C4—H4	120.3	C9—C10—C10^i^	111.85 (14)
C3—C4—H4	120.3	C9—C10—H10B	109.2
C4—C5—C6	121.19 (12)	C10^i^—C10—H10B	109.2
C4—C5—H5	119.4	C9—C10—H10A	109.2
C6—C5—H5	119.4	C10^i^—C10—H10A	109.2
C5—C6—C1	118.56 (11)	H10B—C10—H10A	107.9
C5—C6—C7	119.65 (12)	C7—N1—O2	112.42 (11)
C1—C6—C7	121.79 (11)	C1—O1—H1	109.0 (15)
N1—C7—C6	118.03 (12)	C8—O2—N1	110.45 (10)
N1—C7—H7	122.9 (10)		
			
O1—C1—C2—C3	178.68 (12)	C2—C1—C6—C7	179.53 (12)
C6—C1—C2—C3	−1.30 (19)	C5—C6—C7—N1	175.82 (12)
C1—C2—C3—C4	1.0 (2)	C1—C6—C7—N1	−3.07 (19)
C2—C3—C4—C5	−0.1 (2)	O3—C8—C9—C10	0.66 (19)
C3—C4—C5—C6	−0.6 (2)	O2—C8—C9—C10	179.67 (10)
C4—C5—C6—C1	0.30 (19)	C8—C9—C10—C10^i^	177.66 (13)
C4—C5—C6—C7	−178.63 (12)	C6—C7—N1—O2	−179.06 (10)
O1—C1—C6—C5	−179.35 (12)	O3—C8—O2—N1	−2.57 (18)
C2—C1—C6—C5	0.63 (18)	C9—C8—O2—N1	178.39 (10)
O1—C1—C6—C7	−0.4 (2)	C7—N1—O2—C8	178.03 (11)

Symmetry codes: (i) −*x*+5/2, −*y*+1/2, −*z*+1.

### Hydrogen-bond geometry (Å, °)

**Table:**

*D*—H···*A*	*D*—H	H···*A*	*D*···*A*	*D*—H···*A*
O1—H1···N1	0.91 (3)	1.79 (3)	2.5836 (15)	144 (2)
C4—H4···O1^ii^	0.93	2.45	3.1874 (17)	136
C2—H2···Cg1^iii^	0.93	2.75	3.4659 (14)	134

Symmetry codes: (ii) *x*−1/2, *y*+1/2, *z*; (iii) *x*+1, −*y*−1, *z*−1/2.
